# Mortality in hospitalized SARS-CoV-2 patients with contemporaneous bacterial and fungal infections

**DOI:** 10.1017/ash.2024.424

**Published:** 2024-09-23

**Authors:** John J. Hanna, Zachary M. Most, Lauren N. Cooper, Abdi D. Wakene, Alexander P. Radunsky, Christoph U. Lehmann, Trish M. Perl, Richard J. Medford

**Affiliations:** 1 Information Services, ECU Health, Greenville, NC, USA; 2 Division of Infectious Diseases, Department of Internal Medicine, East Carolina University, Greenville, NC, USA; 3 Clinical Informatics Center, University of Texas Southwestern, Dallas, TX, USA.; 4 Division of Infectious Diseases, Department of Pediatrics, University of Texas Southwestern Medical Center, Dallas, TX, USA; 5 Division of Infectious Diseases & Geographic Medicine, University of Texas Southwestern Medical Center, Dallas, TX, USA; 6 O’Donnell School of Public Health, University of Texas Southwestern, Dallas, TX, USA

## Abstract

**Background:**

The interplay between SARS-CoV-2 and contemporaneous bacterial or fungal culture growth may have crucial implications for clinical outcomes of hospitalized patients. This study aimed to quantify the effect of microbiological culture positivity on mortality among hospitalized patients with SARS-CoV-2.

**Methods:**

In this retrospective cohort study, we included adult hospitalized patients from OPTUM COVID-19 specific data set, who tested positive for SARS-CoV-2 within 14 days of hospitalization between 01/20/2020 and 01/20/2022. We examined outcomes of individuals with organisms growing on cultures from the bloodstream infections (BSIs), urinary tract, and respiratory tract, and a composite of the three sites. We used propensity score matching on covariates included demographics, comorbidities, and hospitalization clinical parameters. In a sensitivity analysis, we included same covariates but excluded critical care variables such as length of stay, intensive care unit stays, mechanical ventilation, and extracorporeal membrane oxygenation.

**Results:**

The cohort included 104,560 SARS-CoV-2 positive adult hospitalized patients across the United States. The unadjusted mortality odds increased significantly with BSIs (98.7%) and with growth on respiratory cultures (RC) (176.6%), but not with growth on urinary cultures (UC). Adjusted analyses showed that BSIs and positive RC independently contribute to mortality, even after accounting for critical care variables.

**Conclusions:**

In SARS-CoV-2-positive hospitalized patients, positive bacterial and fungal microbiological cultures, especially BSIs and RC, are associated with an increased risk of mortality even after accounting for critical care variables associated with disease severity. These findings underscore the importance of stringent infection control and the effective management of secondary infections to improve patient outcomes.

## Introduction

The Coronavirus Disease 2019 (COVID-19) pandemic has underscored the critical need to understand the interplay between viral infections and associated bacterial or fungal infections. Although much attention has been given to the virology of SARS-CoV-2, the prevalence range of contemporaneous bacterial and fungal infections among patients with SARS-CoV-2 has varied greatly across the globe and over the COVID-19 pandemic waves ranging from 1.1% to 45%.^1–33^ Although multiple retrospective studies showed worse outcomes associated with co-infections and secondary infections,^1,2,4,9,17,18,22,25,26^ quantifying the associated positive microbiological culture effect on mortality while accounting for potentially relevant hospitalization events has not been fully explored.

The existing literature on this topic suffers from limitations such as single site cohorts, small sample sizes, lack of control for confounding variables, and/or failure to address the complexity of the interplay between COVID-19 and associated bacterial and fungal infections.^1,2,4,9,11,17,18,22,25,26^ Additionally, clinical interventions, disease severity, and comorbid conditions, which can significantly influence the risk of secondary infections and mortality, have been underreported, leaving an incomplete picture of their contribution to mortality. These research gaps highlight the need for more detailed and rigorous investigations.

Our study aimed to address the above shortcomings and estimate the effect of associated bacterial and fungal infections on mortality in people hospitalized with SARS-CoV-2 infection by employing propensity score matching to balance potentially relevant covariates between patients with and without positive microbiological cultures.

## Methods

### Study design

This retrospective cohort study included adult patients hospitalized within 14 days following a positive SARS-CoV-2 test result between January 20, 2020, and January 20, 2022. The primary outcome was 30-day mortality [defined as mortality occurring in the same calendar month or the calendar month following admission]. A positive microbiologic culture in the first 30 days of hospitalization was the primary exposure of interest. Positive microbiological cultures were used as a proxy for associated bacterial and fungal infections because clinical data needed to confirm a true infection were unavailable. We performed analyses for bloodstream infections (BSI) [defined based on the presence of positive blood culture(s) with any organism(s) within 30 days of admission], growth on urine culture (UC) [Defined based on the presence of any growth of any organism on urinary culture/s within 30 days of admission], growth on respiratory cultures (RC) [Defined based on the presence of any growth of any organism on respiratory culture/s within 30 days of admission], and a combined category of either BSI, growth on UC, or growth on RC. We chose BSI for the primary analysis as BSI-positive microbiological cultures are less likely to represent colonization or contamination compared to other culture types/sources. Two sets of covariates were utilized for propensity score matching and sensitivity analysis resulting in eight distinct analyses of the correlation between microbiological culture positivity and mortality.

### Data collection

We extracted data from the OPTUM COVID-19-specific relational data set, a subset of the certified de-identified national OPTUM longitudinal EHR repository encompassing data from over 700 hospitals and 7,000 clinics across the United States. This data set is laden with a broad spectrum of information including, but not limited to, patient demographics, clinical parameters, healthcare utilization, and outcomes.

### Patient consent statement

We procured the data for this study from the de-identified OPTUM COVID-19 data set, where de-identification was certified by an expert. Under HIPAA, the use of the Expert Determination De-Identification Method allows the reuse of health information without patient authorization. Therefore, our study did not meet our local Institutional Review Board definition of human subject research.

### Covariates

We included an array of covariates that spanned demographic information at the time of admission [age, gender, race/ethnicity, region, insurance type], clinical parameters at the time of admission [BMI, Charlson Comorbidity Index (CCI) score, HIV status, smoking status, time to hospitalization from SARS-CoV-2 positivity], and hospital course details within 30 days of hospitalization [length of stay (LOS), administration of interleukin-6 (IL-6) inhibitors or systemic steroids, central line presence, *Clostridioides difficile* (C.diff) test result, intensive care unit (ICU) stays, mechanical ventilation, and extracorporeal membrane oxygenation (ECMO)].

### Propensity score matching

For each type of infection examined in the analyses, we conducted propensity score matching to account for potential confounding factors between the groups with positive and negative microbiological culture. We executed the matching using the MatchIt package in R,^34^ adopting a nearest-neighbor matching technique with a 1:1 matching ratio. We matched every encounter with a positive microbiological culture to one control encounter devoid of a positive microbiological culture—respecting the type of infection/culture growth examined—based on the closeness of their propensity scores. In a sensitivity analysis, we excluded from the match variables critical care variable such as LOS, mechanical ventilation, ECMO, and ICU stays alongside microbiological culture positivity, since these may potentially be a function of the associated bacterial and fungal infections.

### Statistical analysis

We performed the statistical analyses using RStudio 2023.06.2. For each examined infection type, we calculated unadjusted odds ratios (ORs) and confidence intervals (CIs) for mortality derived from univariate logistic regression models following each propensity score matching.

## Results

### Descriptive statistics by presence of bloodstream infection:

Our study analyzed 104,560 individuals who were hospitalized with SARS-CoV-2 (Table 1) identifying 3,114 (3.0%) cases of BSI. The BSI group was older, with 51% aged 65 or older compared to 42% in the non-BSI group and had a higher proportion of males (62% vs 50%) and African American individuals (19% vs 15%). The BSI group had a higher obesity rate (Class III obesity at 15% vs 13%) and comorbidity burden (median CCI score of 4 vs 1). The time to hospitalization post-SARS-CoV-2 diagnosis was slightly longer for the BSI group. Insurance coverage varied, with Medicare being more common in the BSI group (33% vs 27%), while commercial insurance was less frequent (42% vs 49%). Smokers constituted a slightly larger portion of the BSI group (14% vs 12%).

**Table 1.** Characteristics and outcomes of bloodstream infections (BSIs) (patients with positive blood cultures) among hospitalizations with SARS-CoV-2 infection

Variable	**Overall** N = 104,560^a^	**No BSI** N = 101,446^a^	**BSI** N = 3,114^a^
**Age**			
18–49	32,861/104,560 (31%)	32,298/101,446 (32%)	563/3,114 (18%)
50–64	27,776/104,560 (27%)	26,821/101,446 (26%)	955/3,114 (31%)
65 or older	43,923/104,560 (42%)	42,327/101,446 (42%)	*1,596/3,114 (51%)*
**Gender**			
Female	51,831/104,560 (50%)	50,641/101,446 (50%)	1,190/3,114 (38%)
Male	52,729/104,560 (50%)	50,805/101,446 (50%)	*1,924/3,114 (62%)*
**Race/Ethnicity**			
African American	16,161/104,560 (15%)	15,574/101,446 (15%)	*587/3,114 (19%)*
Asian	2,738/104,560 (2.6%)	2,658/101,446 (2.6%)	80/3,114 (2.6%)
Hispanic White	11,677/104,560 (11%)	11,282/101,446 (11%)	395/3,114 (13%)
Non-Hispanic White	57,235/104,560 (55%)	55,547/101,446 (55%)	1,688/3,114 (54%)
Other	16,749/104,560 (16%)	16,385/101,446 (16%)	364/3,114 (12%)
**Region**			
Midwest	27,926/104,560 (27%)	26,995/101,446 (27%)	*931/3,114 (30%)*
Northeast	20,792/104,560 (20%)	20,189/101,446 (20%)	603/3,114 (19%)
Other/Unknown	6,877/104,560 (6.6%)	6,769/101,446 (6.7%)	108/3,114 (3.5%)
South	43,768/104,560 (42%)	42,431/101,446 (42%)	1,337/3,114 (43%)
West	5,197/104,560 (5.0%)	5,062/101,446 (5.0%)	135/3,114 (4.3%)
**Smoking status**			
Current smoker	12,247/104,560 (12%)	11,815/101,446 (12%)	*432/3,114 (14%)*
Not currently smoking	92,313/104,560 (88%)	89,631/101,446 (88%)	2,682/3,114 (86%)
**BMI**			
Normal Weight	20,862/104,560 (20%)	20,275/101,446 (20%)	587/3,114 (19%)
Obesity Class I	23,813/104,560 (23%)	23,097/101,446 (23%)	716/3,114 (23%)
Obesity Class II	13,964/104,560 (13%)	13,548/101,446 (13%)	416/3,114 (13%)
Obesity Class III	13,643/104,560 (13%)	13,179/101,446 (13%)	*464/3,114 (15%)*
Overweight	30,201/104,560 (29%)	29,355/101,446 (29%)	846/3,114 (27%)
Underweight	2,077/104,560 (2.0%)	1,992/101,446 (2.0%)	85/3,114 (2.7%)
**CCI score**	1.00 (0.00, 4.00)	1.00 (0.00, 4.00)	*4.00 (2.00, 8.00)*
**With HIV**	1,160/104,560 (1.1%)	1,103/101,446 (1.1%)	*57/3,114 (1.8%)*
**Insurance**			
Commercial	50,759/104,560 (49%)	49,440/101,446 (49%)	1,319/3,114 (42%)
Medicaid	13,614/104,560 (13%)	13,197/101,446 (13%)	417/3,114 (13%)
Medicare	28,383/104,560 (27%)	27,345/101,446 (27%)	*1,038/3,114 (33%)*
Other Payor Type	5,086/104,560 (4.9%)	4,924/101,446 (4.9%)	162/3,114 (5.2%)
Uninsured	6,718/104,560 (6.4%)	6,540/101,446 (6.4%)	178/3,114 (5.7%)
**Time to hospitalization**	0.00 (0.00, 1.00)	0.00 (0.00, 1.00)	*0.00 (0.00, 3.00)*
**Received IL6i**	5,520/104,560 (5.3%)	5,182/101,446 (5.1%)	*338/3,114 (11%)*
**Received steroids**	49,031/104,560 (47%)	46,796/101,446 (46%)	*2,235/3,114 (72%)*
**Central lines**	5,656/104,560 (5.4%)	4,812/101,446 (4.7%)	*844/3,114 (27%)*
***Clostridioides Difficile testing***			
Negative	3,109/104,560 (3.0%)	2,784/101,446 (2.7%)	*325/3,114 (10%)*
No test	101,102/104,560 (97%)	98,354/101,446 (97%)	2,748/3,114 (88%)
Positive	349/104,560 (0.3%)	308/101,446 (0.3%)	*41/3,114 (1.3%)*
**Length of stay**	4.00 (2.00, 7.00)	3.00 (2.00, 7.00)	*12.50 (5.00, 24.00)*
**ICU**	18,100/104,560 (17%)	16,545/101,446 (16%)	*1,555/3,114 (50%)*
**ECMO**	228/104,560 (0.2%)	177/101,446 (0.2%)	*51/3,114 (1.6%)*
**Mechanical ventilation**	14,262/104,560 (14%)	12,655/101,446 (12%)	*1,607/3,114 (52%)*
**Death**	9,335/104,560 (8.9%)	8,304/101,446 (8.2%)	*1,031/3,114 (33%)*

a n/N (%); Median (IQR)

The BSI group had a higher proportion of patients receiving steroids (72%) compared to the non-BSI group (46%). Similarly, the administration of IL-6 inhibitors was more prevalent among the BSI group (11%) than non-BSI group (5.1%). Central catheters were used more often in the BSI group, with 27% of patients having had central catheters compared to 4.7% in the non-BSI group. The BSI group exhibited a higher rate of ICU admissions (50%) and mechanical ventilation (52%) compared to the non-BSI group (16% and 12%, respectively). LOS for BSI group was significantly longer, with a median of 12.5 days compared to just 3.0 days for the non-BSI group.

### Mortality outcomes after propensity score matching

**Figure 1.**
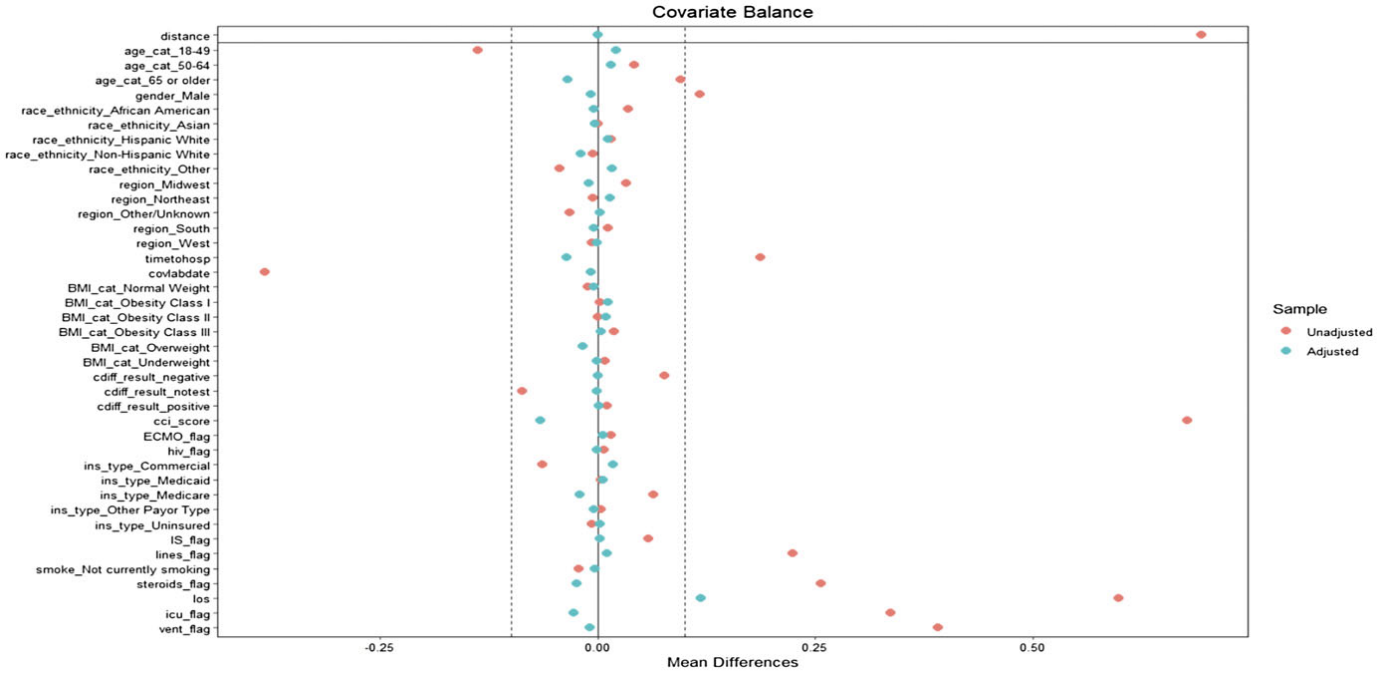
Love plot post-propensity score matching for BSI with all included covariates in the population match.

**Figure 2.**
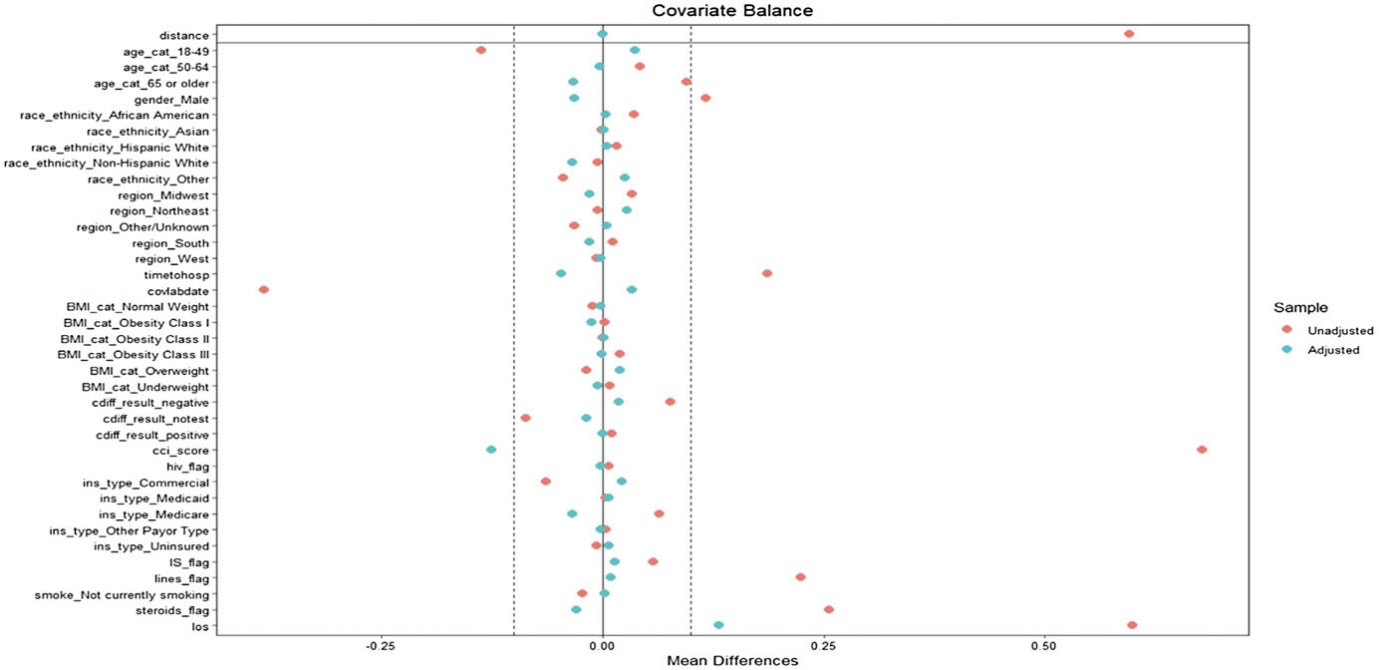
Love plot post-sensitivity analysis propensity score matching for BSI without LOS, ECMO, mechanical ventilation, ICU.

In the primary analysis focusing on BSI, we visualized successful propensity score matching through Love plots (Figure 1 and Figure 2) depicting the balance of covariates between the groups with positive and negative blood cultures. There was a maximum standardized mean difference below the cutoff value of .1 indicating a satisfactory balance of covariates post-matching. Similarly, in the secondary analyses involving growth on UC, growth on RC, and the combined category of either BSI, growth on UC or RC, we also carried out propensity score matching successfully, adhering to the same criteria of covariate balance.

**Figure 3.**
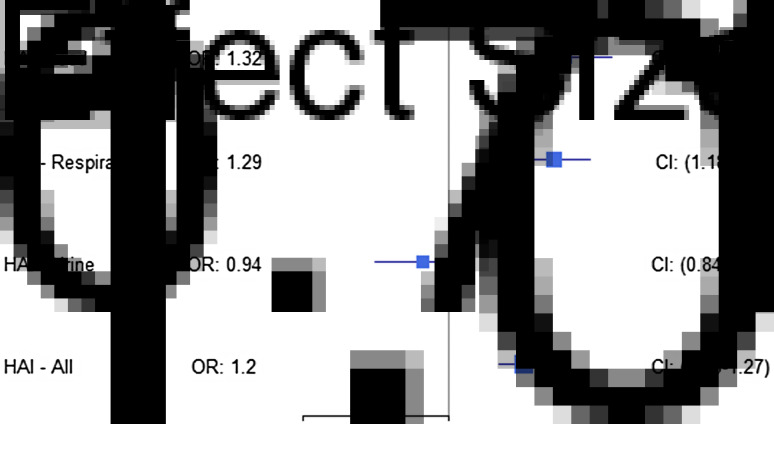
Unadjusted OR for mortality after propensity score matching on all included covariates by type of infection; BSI, respiratory, urine, and all infections.

When we included all variables including LOS, ECMO, ICU, and mechanical ventilation in the propensity score matching (Figure 3), BSI was associated with a 32.3% increase in mortality odds (OR = 1.323, [95% CI, 1.187–1.475]). Growth on RC showed a 28.6% increase in mortality odds (OR = 1.286, [95% CI, 1.180–1.402]). Growth on UC was not associated with a significant increase in mortality odds (OR = .939, [95% CI, .838–1.053]). For all associated bacterial and fungal infections/colonization combined, the mortality odds increased by 19.6% (OR = 1.196, [95% CI, 1.128–1.268]).

**Figure 4.**
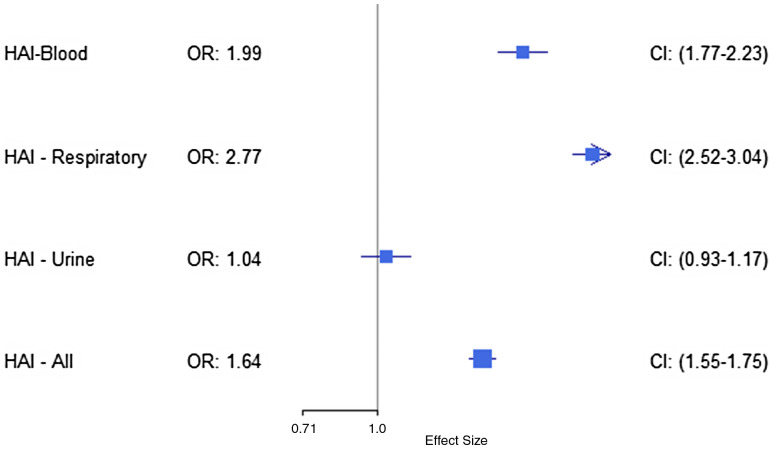
Unadjusted OR for mortality after propensity score matching on all covariates except LOS, mechanical ventilation, ICU, and ECMO by type of infection; BSI, respiratory, urine, and all infections.

In the sensitivity analysis statistical models that excluded LOS, ICU, mechanical ventilation, and ECMO from the propensity scores (Figure 4), the unadjusted OR for mortality showed that patients with BSI had 98.7% greater mortality odds compared to those without (OR = 1.987, [95% CI, 1.771–2.231]). Growth on RC was associated with a 176.6% increase in mortality odds (OR = 2.766, [95% CI, 2.520–3.038]). Growth on UC again did not show a statistically significant increase in mortality odds (OR = 1.043, [95% CI, .929–1.172]). When considering all associated bacterial and fungal infections/colonizations together, we found a 64.4% increase in mortality odds (OR = 1.644, [95% CI, 1.548–1.746]).

## Discussion

Our comprehensive study into the role of associated bacterial and fungal infections/colonization among hospitalized COVID-19 patients observed that growth of bacteria or fungi from blood cultures or respiratory tract cultures were significantly associated with higher mortality odds. Conversely, growth from urine cultures did not demonstrate a statistically significant effect on mortality odds, which may reflect lower risk of uncomplicated urinary tract infections (UTIs) and the complex nature of labeling the clinical significance of UC that are often clouded by colonization and contamination issues.

The study’s descriptive statistics highlight that BSIs occur in older individuals with more comorbidities. This suggests that BSIs in the context of COVID-19 may not merely be opportunistic but could also be indicative of the overall health trajectory of these patients. The preponderance of BSIs in older and more comorbid patients aligns with existing literature that associates advanced age and pre-existing health conditions with increased susceptibility to infections and worse outcomes.^1,2,10^ The gender and racial disparities we observed—with a higher proportion of males and African American patients in the BSI-positive group—point to potential biological and socioeconomic factors influencing infection risks and outcomes and an existing health inequality.

The heightened mortality risk associated with BSIs and RC positivity is consistent with findings in prior studies, which documented the severe effect of such co-infections on patient outcomes in the context of viral pandemics.^1,2,4,9,17,18,22,25,26^ As contemporaneous infections may contribute to ICU stay, increased LOS, mechanical ventilation, ECMO, and consequently death, we performed a sensitivity analysis that did not include these variables in the model. When these variables were excluded from matching, the odds of mortality significantly increased for patients with BSIs and with growth on RC. The design of our study not only allowed us to validate findings from previous studies, but also to quantify the difference in mortality odds before and after accounting for critical care measures that reflect clinical disease severity potentially due to the severity of COVID-19 or the severity of the associated infection.

Compared to non-intubated patients, in addition to higher likelihood of worse outcomes and higher rates of respiratory track colonization, intubated patients are also subject to a higher number of RC collections. When we initially employed propensity score matching on critical care variables including mechanical ventilation, we aimed to account for indication bias between those with culture growth and those without. However, it is important to consider the increased risk of mortality associated with intubated critically ill patients with a respiratory infection by itself while interpreting the reported OR associated with growth on RC. This potentially explains the inflated odds of mortality associated with growth on RC when we excluded critical care variables like mechanical ventilation from the propensity score match in the sensitivity analysis.

In our analysis, 15% of included individuals with central lines had at least one positive blood culture, and only 27% of patients with presumed BSI had an associated central line, raising the question of source of BSI in the remaining majority. Although missingness is one of the inherent limitations of using real world data, this relatively low association with catheter use, if real, may suggest an alternative source of BSI or potentially higher rates of blood culture contamination in the patient population.

Our study demonstrates that the occurrence of positive microbiological cultures in patients hospitalized with SARS-CoV-2 is associated with an increased mortality risk, which persists beyond the confounding effects of clinical severity and the more frequent testing of critically ill patients. This elevated risk is evident even after adjusting for the therapeutic interventions for COVID-19, such as systemic steroids and IL-6 inhibitors, and after accounting for the use of invasive procedures like central lines, mechanical ventilation, and ECMO using propensity score matching. These findings signal that contemporaneous bacterial and fungal infections are likely to be substantial contributors to mortality, which accentuates the critical role of meticulous infection control and the judicious use of therapeutic and supportive interventions in the COVID-19 treatment paradigm to diminish the added mortality burden of secondary infections.

### Limitations

The incidental inclusion of individuals hospitalized with asymptomatic SARS-CoV-2 positivity for other reasons could skew the association between secondary infections in the context of COVID-19 disease and mortality. Although positive microbiological culture results may not represent true infections, the incidence reported in our study would be more reflective of positive microbiological cultures during SARS-CoV-2 positive hospitalizations rather than the incidence of SARS-CoV-2 associated infections. Additionally, our inability to differentiate from the data between colonization, contamination, and true infection may have overestimated or underestimated the association between positive microbiological cultures and mortality. The reliance on a de-identified data set also meant that the precise timing of death could not be ascertained, necessitating a reliance on the assumption that death occurred in the month of or the month following hospitalization would be included in the 30-day mortality definition. Lastly, considering we used a COVID-19 specific data set, and the study question focused on hospitalization among SARS-CoV-2 positive patients, we were unable to compare mortality among hospitalized patients with SARS-CoV-2 and those without SARS-CoV-2. These factors, while inherent in many large-scale epidemiological studies, must be carefully weighed when interpreting the results. However, the large sample size of our study and the application of rigorous dual propensity score matching technique served to mitigate these issues and deliver credible population-level insights.

### Future work

Future research should aim to refine the understanding of associated bacterial and fungal infections in patients with COVID-19 by focusing on prospective data collection, which allows for better control over the timing and characterization of infections. Studies could also benefit from a more detailed analysis of the effect of individual critical care interventions and their timing relative to the onset of secondary infection. Additionally, there is a need to develop methods that more accurately distinguish between true infections and other forms of positive microbiological test results, such as colonization or contamination when utilizing real world data or EHR data in research. Further investigations into the demographic disparities observed could elucidate underlying causes and inform strategies to address inequalities.

## Conclusion

Our large US national study adds to the growing evidence that associated bacterial and fungal infections, particularly those causing BSIs and growth on RC, are associated with significantly increased mortality odds in hospitalized patients with SARS-CoV-2 infection. The findings of our study underscore the importance of vigilant prevention, monitoring, and management of these infections to improve patient outcomes.
